# Editorial Changes

**Published:** 1849-12

**Authors:** 


*Editorial Changes.*—The Charleston (S. C.) Medical Journal and Review for November, 1849, contains the announcement that the connection between it and its former Editors closes with that number. Drs. Gaillard and DeSaussure are suceeded by Drs. J. C. Cain and E. F. Porcher; names known to medical literature from their valuable communications for the pages of the Journal, the charge of which they are now to assume. The Charleston Journal hitherto has been conducted with signal ability, and its reputation, we trust, will not deteriorate by passing into new hands.

The above announcement suggested the inquiry, 'how many of our American contemporaries have continued without Editorial changes during the period that has elapsed since the Buffalo Journal was commenced, now nearly five years?' On referring to our list of exchanges in the United States, we enumerate, in all 12. Of these but *three* remain in the editorial hands in which they were when we entered on Editorial labors. The unchanged Journals are, the American Journal of Medical Sciences, including the Medical News and Library; the Boston Medical and Surgical Journal, and the New Jersey Medical Reporter, the latter having been established about two years. During the period referred to, at least half a dozen medical periodicals have been discontinued. These statistics exemplify the uncertainty, or instability of medical Editorship in this country; and are not without a significancy which we leave, for the present, to the surmise of the reader.

